# 超高效合相色谱-串联质谱法快速检测药用复合膜中12种光引发剂残留

**DOI:** 10.3724/SP.J.1123.2025.03025

**Published:** 2025-10-08

**Authors:** Yunjiao CAO, Chenxi LIU, Leilin CHEN, Yue SHANG, Xiaoli CHEN, Bei HU, Meng FU, Min HU

**Affiliations:** 1.湖北中医药大学药学院，湖北 武汉 430065; 1. School of Pharmacy，Hubei University of Chinese Medicine，Wuhan 430065，China; 2.湖北省药品监督检验研究院，湖北省药品质量检测与控制;工程技术研究中心，国家药品监督管理局中药质量控制重点实验室，湖北 武汉 430075; 2. Hubei Institute for Drug Control，Hubei Engineering Research Center for Drug Quality Control，National Medical Products Administration;Key Laboratory of Traditional Chinese Medicine，Wuhan 430075，China; 3.国家药典委员会，北京 100061; 3. Chinese Pharmacopoeia Commission，Beijing 100061，China

**Keywords:** 超高效合相色谱-串联质谱, 超临界CO_2_, 药用复合膜, 光引发剂, ultra performance convergence chromatography-tandem mass spectrometry （UPC^2^-MS/MS）, supercritical CO_2_, medicinal composite membranes, photoinitiators

## Abstract

建立了超高效合相色谱-串联质谱（UPC^2^-MS/MS）快速测定药用复合膜中12种光引发剂残留量的方法。以乙腈提取药用复合膜中残留的光引发剂。采用ACQUITY UPC^2 ^CSH^TM^ Fluoro-Phenyl色谱柱（100 mm×3.0 mm， 1.7 μm）进行分离，以超临界二氧化碳-甲醇为流动相进行梯度洗脱，以甲醇-水（99∶1，v/v）为补偿液。流动相流速为1.5 mL/min，补偿液流速为0.2 mL/min，系统背压为13.79 MPa，柱温为50 ℃，进样量为1 μL。采用电喷雾（ESI）离子源，在正离子多反应监测（MRM）模式下检测12种光引发剂的离子对，外标法定量。结果表明，12种光引发剂在0.1~2.0 μg/mL范围内具有良好的线性关系，相关系数（*r*）> 0.995，检出限（LOD）均为0.03 μg/mL，定量限（LOQ）均为0.1 μg/mL。12种光引发剂在3个加标水平下的加标回收率为80.7%~119.7%，相对标准偏差为1.0%~5.6%。采用该方法对12批药用复合膜进行测定，共发现6批阳性样品，检出的4种光引发剂分别为4-甲基二苯甲酮、邻苯甲酰苯甲酸甲酯、二苯甲酮和2-异丙基硫杂蒽酮，其中邻苯甲酰苯甲酸甲酯的检出量最高，未超过拟定限度。本方法准确、灵敏、快速、环保，样品前处理简便，适用于监测药用复合膜中光引发剂的残留量，能为药品包装材料质量标准的完善提供研究基础。

## Abstract (en)

Photoinitiators are indispensable components in the formulation of inks used for printing packaging materials. Residual photoinitiators on the surface of packaging materials may migrate and contaminate the contents， posing potential risks to human health. Toxicological experiments have shown that photoinitiators pose various risks， including carcinogenicity， reproductive toxicity， and dermal toxicity. However， current quality standards for pharmaceutical packaging materials， both domestically and internationally， do not impose limits on photoinitiators. This study focuses on 12 photoinitiators， which are either restricted or prohibited in certain fields such as food packaging materials. The selected medicinal composite membranes， widely employed in pharmaceutical packaging and requiring significant ink volumes， serve as representative samples. This research holds significant implications for improving the quality standards of pharmaceutical packaging materials and ensuring medication safety. While gas chromatography-mass spectrometry and ultra performance liquid chromatography-tandem mass spectrometry have been reported for photoinitiators detection， UPC^2^-MS/MS has not yet been reported for detecting these compounds. The principle of UPC^2^ is based on supercritical fluid chromatography， where the mobile phase primarily consists of supercritical CO_2_ with a minimal use of organic solvents， aligning with the trend of green chemistry. When coupled with MS， this method also enhances the sensitivity of detection. Therefore， a novel method based on UPC^2^-MS/MS was established for the determination of the 12 photoinitiator residues in medicinal composite membranes. MS conditions， makeup solution conditions （solvent， additive type， additive ratio， flow rate）， and sample pretreatments were systematically optimized. Photoinitiator residues were extracted from the medicinal composite membranes using acetonitrile by ultrasonication. The chromatographic separation was achieved on a UPC^2^ CSH^TM^ Fluoro-Phenyl column （100 mm × 3.0 mm， 1.7 μm） under gradient elution using a mobile phase composed of supercritical carbon dioxide （A） and methanol （B）. Gradient elution was performed as follows： 0-1.5 min， 100%A-95%A； 1.5-2.0 min， 95%A-80%A； 2.0-3.0 min， 80%A-70%A； 3.0-4.0 min， 70%A-100%A； 4.0-5.0 min， 100%A. A methanol-water （99∶1， v/v） mixture was employed as the makeup solution to enhance MS response. The mobile phase flow rate， makeup solution flow rate， column temperature， and injection volume were respectively set at 1.5 mL/min， 0.2 mL/min， 50 ℃ and 1 μL. The automatic back pressure regulator maintained 13.79 MPa. The 12 photoinitiators were monitored in multiple reaction monitoring （MRM） mode with electrospray ionization （ESI） under positive conditions. Quantification was performed using the external standard method. The results showed that all 12 photoinitiators exhibited good linear relationships in the range of 0.1-2.0 μg/mL， with correlation coefficients （*r*）>0.995. The limits of detection （LODs） and quantification （LOQs） for the 12 photoinitiators were 0.03 μg/mL and 0.1 μg/mL， respectively. At 1LOQ， 2LOQ， and 10LOQ levels， the average recoveries of the 12 photoinitiators ranged from 80.7% to 119.7%， with relative standard deviations （RSDs） between 1.0% and 5.6%. Twelve batches of medicinal composite membranes were tested using this method， and six batches were found positive. The detected photoinitiators were 4-methylbenzophenone， methyl-2-benzoylbenzoate， benzophenone， and 2-isopropylthioxanthone. Among them， methyl-2-benzoylbenzoate showed the highest concentration， but it did not exceed the proposed limit. The established method is accurate， sensitive， rapid， environmentally friendly， and simple pretreatment， which is suitable for monitoring residual photoinitiators in medicinal composite membranes.

光引发剂是使用油墨印刷包装材料时必需的配方成分^［1］^。残留在包装材料中的光引发剂会通过物理接触或者化学迁移的方式对人体健康造成潜在危害，如光引发剂二苯甲酮可能会引起皮肤过敏，甚至致癌^［2］^。欧盟明确规定光引发剂四乙基米氏酮和米氏酮禁用于食品包装材料^［3］^；瑞士将光引发剂4-甲基二苯甲酮列入食品包装A类管控清单^［4］^；中国亦对食品包装中光引发剂的使用实施限制^［5］^。然而在药品包装材料领域，国内外尚未建立光引发剂使用规范，尤其是对油墨使用量较大、应用较广泛的药用复合膜材料。因此，建立药用复合膜中光引发剂残留量的监测方法，对完善行业质量标准和保障用药安全具有重要意义^［6］^。

文献中主要采用气相色谱-质谱法^ ［7-12］^、液相色谱-质谱法^［13-15］^、高效液相色谱法^［16］^等检测光引发剂，未见采用超高效合相色谱-质谱法检测光引发剂的报道。超高效合相色谱^［17，18］^的流动相主要是超临界CO_2_，有机溶剂使用量较少，符合绿色化学的趋势，其与质谱联用后还可进一步提升检测方法的灵敏度^［19］^。

本研究选取食品包装中有限定要求或者禁用的12种光引发剂为目标分析物，基于超高效合相色谱-串联质谱（UPC^2^-MS/MS）技术，通过优化样品前处理条件、质谱参数、补偿液条件等，建立了一种高灵敏度的定量分析方法，可用于检测药用复合膜中残留水平较低的光引发剂。

## 1 实验部分

### 1.1 仪器与试药

ACQUITY^TM^型超高效合相色谱仪和Xevo-TQD三重四极杆质谱仪（美国Waters公司）；Hyper Sonic DT-A超声波清洗器（鼎泰恒盛科技有限公司）；XP205分析天平（瑞士梅特勒-托利多）；CO_2_（武汉市明辉气体科技有限公司，含量：99.999%）；甲醇、乙醇、乙腈、二氯甲烷（色谱纯，德国Merck公司）；甲酸（色谱纯，美国ACS Lab公司）。

12种光引发剂均购于上海麦克林公司（纯度均>98%），具体信息：二苯甲酮（BP）、对-*N，N*-二甲氨基苯甲酸乙酯（EDB）、4-甲基二苯甲酮（4-MBP）、3，4-二甲基二苯甲酮（3，4-DBP）、对二甲氨基苯甲酸异辛酯（EHDAB）、2-异丙基硫杂蒽酮（2-ITX）、2，4-二乙基硫杂蒽酮（DETX）、2-甲基二苯甲酮（2-MBP）、3-甲基二苯甲酮（3-MBP）、邻苯甲酰苯甲酸甲酯（OMBB）、四乙基米氏酮（DEAB）、米氏酮（MK）。12批药用复合膜，具体信息见表1。

**表1:** 药用复合膜信息

No.	Sample name	Sample materials	Manufacturer	Batch No.
Y01	Calcium Carbonate and Vitamin D_3_ Granules	polyester/aluminum/polyethylene	A	230501
Y02	Calcium Carbonate and Vitamin D_3_ Granules	polyester/aluminum/polyethylene	B	240502
Y03	Calcium Carbonate and Vitamin D_3_ Granules	paper/aluminum/polyethylene	C	231211
Y04	Calcium Carbonate and Vitamin D_3_ Granules	paper/aluminum/polyethylene	C	231110
Y05	Calcium Carbonate and Vitamin D_3_ Granules	paper/aluminum/polyethylene	D	3H239002
Y06	Calcium Carbonate and Vitamin D_3_ Granules	paper/aluminum/polyethylene	D	3H239003
Y07	Calcium Carbonate and Vitamin D_3_ Granules	polyester/aluminum/polyethylene	E	240102
Y08	Calcium Carbonate and Vitamin D_3_ Granules	polyester/aluminum/polyethylene	E	230504
Y09	Calcium Carbonate and Granules	polyester/aluminum/polyethylene	F	20231108
Y10	Calcium Carbonate and Granules	polyester/aluminum/polyethylene	G	2303014
Y11	Oseltamivir Phosphate Granules	polyester/aluminum/polyethylene	I	2304128
Y12	Oseltamivir Phosphate Granules	polyester/aluminum/polyethylene	J	2403048

### 1.2 对照品溶液与供试品溶液的制备

对照品溶液：精密称取12种光引发剂各100 mg，置于100 mL棕色容量瓶中，加入10 mL乙腈溶解后，定容至刻度，摇匀，即得到1 mg/mL的混合对照品储备液A；精密量取10 mL储备液A，置于100 mL棕色容量瓶中，用乙腈稀释、定容至刻度，摇匀，即得100 μg/mL的混合对照品溶液①；精密量取1 mL储备液A，置于100 mL棕色容量瓶中，加入乙腈稀释、定容至刻度，摇匀，配制成10 μg/mL的混合对照品溶液②；精密量取1 mL混合对照品溶液①，置于100 mL棕色容量瓶中，用乙腈稀释、定容至刻度，摇匀，即得1.0 μg/mL的混合对照品溶液③。

供试品溶液：取药用复合膜样品90 cm^2^，将其裁剪成0.5 cm×0.5 cm的碎片，置于50 mL具塞锥形瓶中，精密加入10 mL乙腈，密塞，超声（功率800 W，频率40 kHz）提取30 min，按需补足失重，经0.22 μm有机膜过滤，取续滤液，即得。

### 1.3 UPC^2^-MS/MS条件

UPC^2^参数：ACQUITY UPC^2^ CSH^TM^ Fluoro-Phenyl色谱柱（100 mm×3.0 mm， 1.7 μm）；流动相A为超临界CO_2_，流动相B为甲醇，梯度洗脱程序：0~1.5 min，100%A~95%A；1.5~2.0 min，95%A~80%A；2.0~3.0 min，80%A~70%A；3.0~4.0 min，70%A~100%A；4.0~5.0 min，100%A。流动相流速为1.5 mL/min；系统背压为13.79 MPa；柱温为50 ℃；进样量为1 μL。

质谱参数：电喷雾电离源， 正离子（ESI^+^），多反应监测（MRM）模式；离子源温度为150 ℃；去溶剂气流速为800 L/h，温度为500 ℃；毛细管电压为3.2 kV；锥孔气流速为50 L/h；补偿液为甲醇-水（99∶1，v/v），补偿液流速为0.2 mL/min；12种光引发剂的保留时间、离子对、锥孔电压（CV）、碰撞能量（CE）等质谱参数见表2。

**表2:** 12种光引发剂的MS参数

Compound	Abbreviation	CAS No.	Molecular formula	Retention time/min	Parent ion （*m*/*z*）	CV/ V	Daughter ion （ *m/z*）	CE/ eV	Chemical structure
Benzophenone	BP	119-61-9	C_13_H_10_O	1.33	183.0	30	105.0^*^ 77.0	25 35	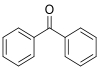
Ethyl 4-dimethylaminobenzoate	EDB	10287-53-3	C_11_H_15_NO_2_	1.70	194.1	30	151.1^*^ 166.0	35 25	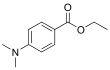
4-Methybenphenone	4-MBP	134-84-9	C_14_H_12_O	1.44	197.1	30	105.1^*^ 77.1	25 35	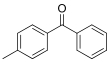
3，4-Dimethylbenzophenone	3，4-DBP	2571-39-3	C_15_H_14_O	1.54	211.1	30	105.1^*^ 77.1	25 40	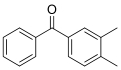
2-Ethylhexyl 4-（dimethylamino）benzoate	EHDAB	21245-02-3	C_17_H_27_NO_2_	1.64	278.0	30	151.1^*^ 166.0	45 30	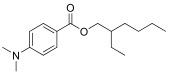
2-Isopropylthioxanthone	2-ITX	5495-84-1	C_16_H_14_OS	1.84	255.1	40	212.9^*^ 184.0	30 55	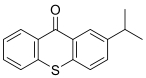
2，4-Diethyl-9*H*-thioxanthen-9-one	DETX	82799-44-8	C_17_H_16_OS	2.24	269.0	40	212.9^*^ 184.0	30 55	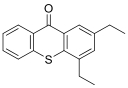
2-Methybenphenone	2-MBP	131-58-8	C_14_H_12_O	1.25	197.1	30	105.1^*^ 77.1	20 40	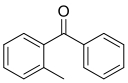
3-Methybenphenone	3-MBP	643-65-2	C_14_H_12_O	1.37	197.1	30	105.1^*^ 77.1	25 25	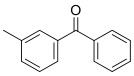
Methyl-2-benzoylbenzoate	OMBB	606-28-0	C_15_H_12_O_3_	1.60	241.0	30	209.1^*^ 152.0	20 50	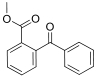
4，4′-Bis（diethylamino）benzopheone	DEAB	90-93-7	C_21_H_28_N_2_O	2.81	325.2	30	176.0^*^ 281.0	40 40	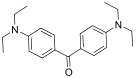
Micher’s ketone	MK	90-94-8	C_17_H_20_N_2_O	2.90	269.1	40	148.0^*^ 91.0	35 40	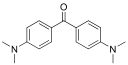

CV： cone voltage； CE： collision energy； * quantitative ion.

## 2 结果与讨论

### 2.1 UPC^2^色谱条件的选择

前期研究对UPC^2^的色谱参数进行摸索，发现色谱柱种类、柱温、改性剂、系统背压等均会影响化合物的分离效果，其中色谱柱的填料与改性剂种类主要影响选择性，柱温和系统背压会通过改变主流动相超临界二氧化碳的密度从而影响分离效果，优化后的参数详见1.3节。在优化条件下，对12种光引发剂的混合对照品溶液（1.0 μg/mL）进行测定，其总离子流色谱图见图1。

**图1:**
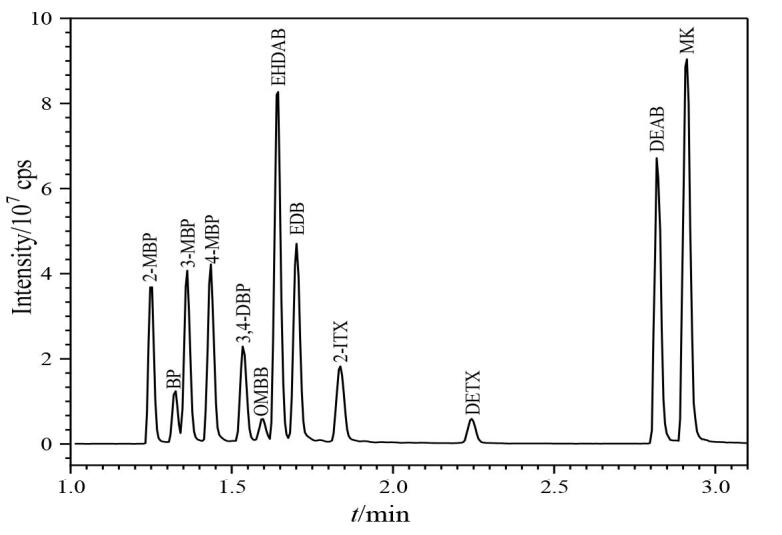
12种光引发剂混合标准溶液（1.0 μg/mL）的总离子流色谱图

### 2.2 质谱条件的选择

将12种光引发剂用补偿液稀释至适当浓度后依次注入离子源，在ESI^+^下进行一级质谱全扫描均获得［M+H］^+^母离子；再调整CV，进行二级质谱扫描，取响应值高、干扰小的2个碎片离子作为子离子（响应较高的离子作为定量离子），并优化CE，经优化后得到的质谱参数见表2。

### 2.3 补偿液的选择

UPC^2^系统中的流动相主要是超临界CO_2_。而当CO_2_流经背压调节器之后的管路时，会因减压作用从超临界态缩变成气态，化合物电离所需的溶液基质较少^［20］^，导致目标物难以有效电离，进而造成质谱响应低甚至无响应。为改善此状况，需要在柱后添加补偿液以促进目标物的电离，增强质谱响应。因此，补偿液的组成对目标分析物的质谱响应具有较大的影响。

#### 2.3.1 补偿液中溶剂种类的选择

实验考察了补偿液中溶剂种类（甲醇、乙醇）对12种光引发剂质谱响应的影响。以乙醇作为溶剂时定量离子的峰面积为参考，将甲醇作为溶剂时的峰面积与之相比作图，得到12种光引发剂在不同溶剂下的相对响应值。

如图2所示，当使用甲醇替代乙醇作为补偿液的溶剂时，有8种光引发剂的质谱响应显著增强，因此选择甲醇作为补偿液的溶剂。多数光引发剂在甲醇作为补偿液的溶剂时质谱响应增加，可能是因为甲醇比乙醇黏度低，流动性强，更容易获得较好的质谱响应；还可能是因为甲醇的酸性比乙醇强，更容易电离出氢离子，促进了大部分光引发剂的电离，增强了质谱响应。

**图2:**
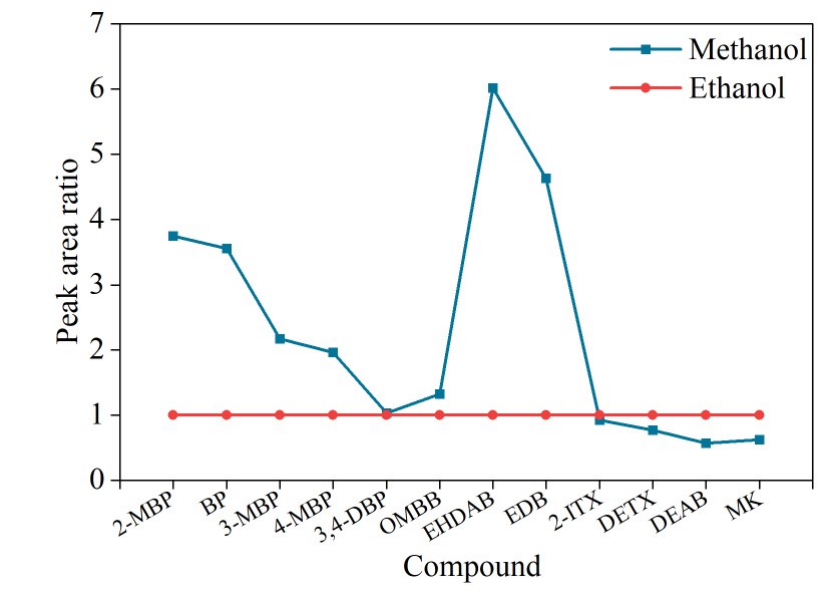
补偿液中溶剂种类对12种光引发剂质谱响应的影响

#### 2.3.2 补偿液中添加剂种类的选择

实验考察了补偿液中添加剂种类（甲酸、水）对12种光引发剂质谱响应的影响。以纯甲醇作为补偿液时定量离子的峰面积为参考，将甲酸、水作为补偿液中添加剂时的峰面积与之相比作图，得到12种光引发剂在不同添加剂下的相对响应值。

如图3所示，当使用甲酸作为补偿液的添加剂时，有9种光引发剂的质谱响应降低，说明甲酸对大多数光引发剂的电离有一定抑制作用，从而影响质谱响应。谢婷婷^［21］^在研究添加剂种类对硫双威质谱响应的影响时，亦发现甲酸降低目标分析物的质谱响应。当使用水作为补偿液的添加剂时，12种光引发剂的质谱响应均增加，说明水可以促进光引发剂电离。因此选择水作为补偿液的添加剂。

**图3:**
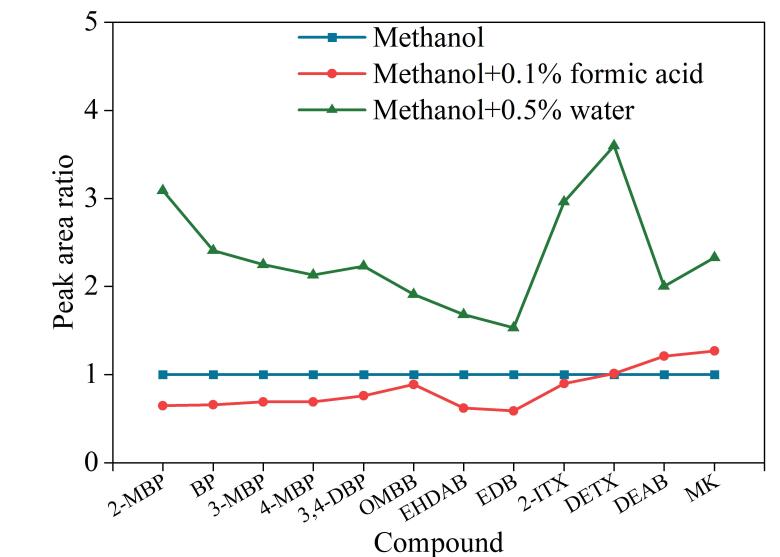
补偿液中添加剂种类对12种光引发剂质谱响应的影响

#### 2.3.3 补偿液中水体积分数的选择

实验还考察了补偿液中水的体积分数（0.5%、1%、2%、3%、5%）对12种光引发剂质谱响应的影响。以水的体积分数为0.5%时定量离子的峰面积为参考，将水的体积分数为1%、2%时的峰面积与之相比作图，得到12种光引发剂在不同水体积分数下的相对响应值。

如图4所示，当补偿液中水的体积分数为0.5%时，12种光引发剂的质谱响应均明显低于水的体积分数为1%、2%时的质谱响应。当补偿液中水的体积分数为1%时，12种光引发剂中有6种的质谱响应高于水的体积分数为2% 时的质谱响应，其余6种光引发剂则表现出相反的趋势。此外，实验发现当补偿液中水的体积分数超过3%时，仪器状态不稳定，这可能是因为水具有高比热容，这一特性会影响系统动态背压阀的控温稳定性，进而导致管路中的CO₂难以维持在超临界流体状态，或者形成双相流动相^［22］^。因此，基于对较低水含量的优先考虑，将补偿液中水的体积分数定为1%。

**图4:**
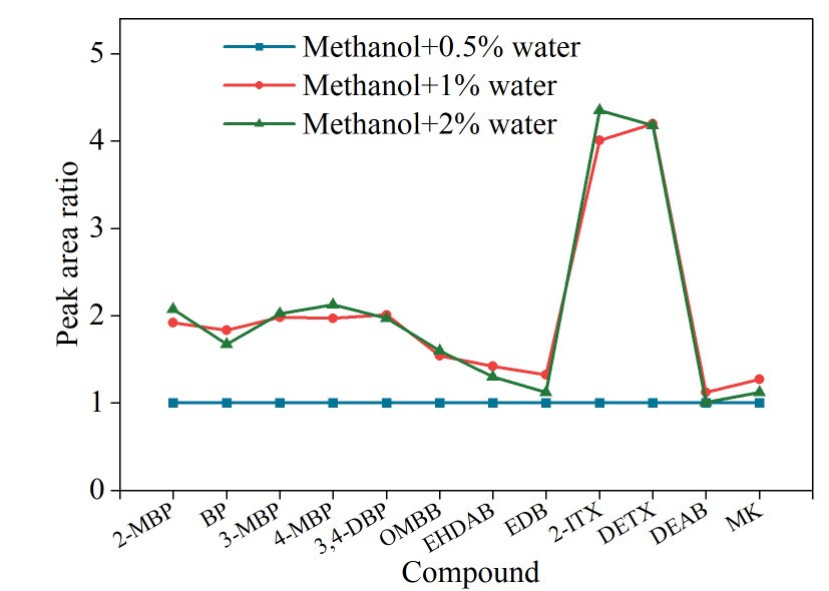
补偿液中水的体积分数对12种光引发剂质谱响应的影响

#### 2.3.4 补偿液流速的选择

实验考察了补偿液流速（0.1、0.2、0.3、0.4、0.5、0.6、0.8 mL/min）对12种光引发剂质谱响应的影响。结果表明，0.1 mL/min的流速过低，质谱几乎无信号响应。以0.2 mL/min流速时定量离子的峰面积为参考，将在0.3~0.8 mL/min流速下的峰面积与之相比作图，得到12种光引发剂在不同补偿液流速下的相对响应值。

如图5所示，当补偿液流速为0.2 mL/min时，12种光引发剂的质谱响应最高，继续增加补偿液的流速，质谱响应不断降低。这可能是补偿液流速过大后，系统压力增大迫使背压阀开启排放功能^［23］^，分流了部分光引发剂，进而导致质谱响应较低。因此，补偿液的流速选择0.2 mL/min。

**图5:**
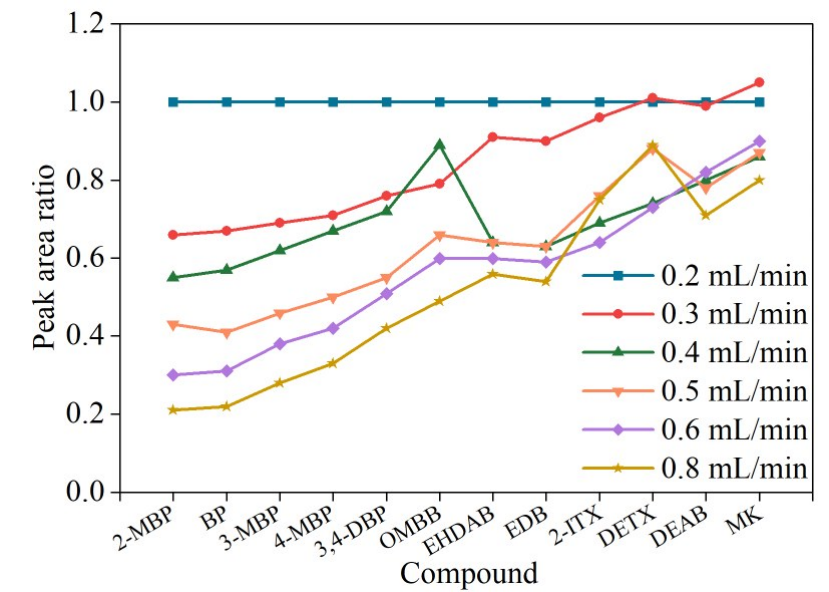
补偿液流速对12种光引发剂质谱响应的影响

### 2.4 样品前处理条件的优化

预实验中Y10药用复合膜样品检测出的光引发剂种类最多（3种），选取该批样品考察了乙腈、二氯甲烷对光引发剂提取效果的影响，发现乙腈提取光引发剂时的干扰相对较少，与参考文献［^24^］一致。实验考察了超声提取时间为10、20、30、40 min时对光引发剂提取效果的影响。结果如图6所示，超声30 min后药用复合膜中的光引发剂提取量趋于稳定。因此，选择30 min超声处理时间进行样品提取。

**图6:**
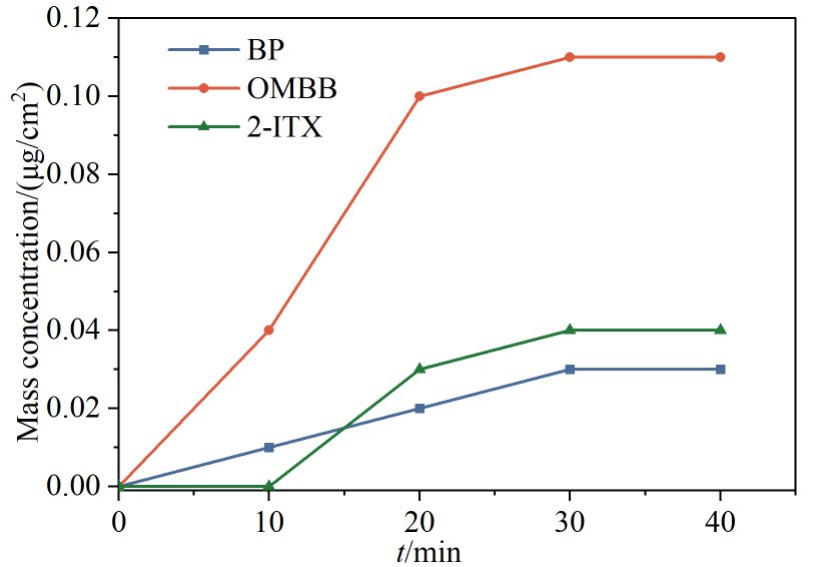
超声时间对药用复合膜中光引发剂提取效果的影响

### 2.5 方法学验证

#### 2.5.1 线性范围、检出限与定量限

精密量取0.1、0.25、0.5、1.0、2 mL的混合对照品溶液②，分别置于10 mL棕色容量瓶中，用乙腈稀释、定容、摇匀，得到0.1、0.25、0.5、1.0、2.0 μg/mL系列标准溶液，0.22 μm有机滤膜过滤，取续滤液，按照1.3节进行检测。以12种光引发剂的峰面积（*y*）为纵坐标，以质量浓度（*x*，μg/mL）为横坐标，得到线性方程。将混合对照品溶液③进行逐级稀释测量，信噪比约为3时得到检出限，约为10时得到定量限。结果见表3，12种光引发剂在0.1～2.0 μg/mL范围内，线性关系均良好（*r>*0.995）。

**表3:** 12种光引发剂线性关系、检出限、定量限及精密度

Compound	Linear equation	*r*	LOQ/（μg/mL）	LOD/（μg/mL）	RSD/% （*n*=6）
2-MBP	*y*=4110.0*x*-50.0	0.9987	0.1	0.03	2.5
BP	*y*=5353.7*x*+46.5	0.9997	0.1	0.03	2.4
3-MBP	*y*=19288.0*x*-38.0	0.9998	0.1	0.03	2.0
4-MBP	*y*=5273.2*x*+155.0	0.9996	0.1	0.03	3.3
3，4-DBP	*y*=39454.1*x*-121.4	0.9990	0.1	0.03	3.4
OMBB	*y*=9712.83*x*-190.9	0.9954	0.1	0.03	3.4
EHDAB	*y*=158902.0*x*-1409.4	0.9996	0.1	0.03	1.1
EDB	*y*=84248.6*x*+289.8	0.9984	0.1	0.03	1.3
2-ITX	*y*=17942.8*x*-313.5	0.9992	0.1	0.03	4.0
DETX	*y*=1095.4*x*-41.4	0.9991	0.1	0.03	3.5
DEAB	*y*=11554.6*x*+877.9	0.9958	0.1	0.03	3.9
MK	*y*=5548.1*x*+391.2	0.9961	0.1	0.03	0.7

*y*：peak area； *x*： mass concentration，μg/mL. Linear range： 0.1-2.0 μg/mL.

#### 2.5.2 重复性

将混合对照品溶液③，按照1.3节进行检测，连续进样6次，记录定量离子的峰面积，计算RSD为0.7%~4.0%，说明该法重复性良好，具体结果见表3。

#### 2.5.3 加标回收率

以预实验中光引发剂阴性的药用复合膜（样品编号：Y01）作为空白基质样品；裁取90 cm^2^阴性样品3份，分别向其中精密加入10、20、100 μL的混合对照品储备液①，按照1.2节的供试品制备方法处理，得到1LOQ、2LOQ、10LOQ的3个加标水平的溶液，每个加标水平重复测定6次，按照1.3节进行检测。

如表4所示，12种光引发剂的加标回收率为80.7%~119.7%，相对标准偏差为1.0%~5.6%，说明该方法的准确度良好。

**表4:** 12种光引发剂的回收率和相对标准偏差（*n*=6）

Compound	1LOQ		2LOQ		10LOQ	
	Recovery/%	RSD/%	Recovery/%	RSD/%	Recovery/%	RSD/%
2-MBP	112.8	5.6	110.4	3.4	118.7	2.3
BP	92.0	5.0	80.7	2.9	102.9	1.8
3-MBP	85.0	1.9	84.8	2.6	106.6	2.2
4-MBP	91.9	3.0	88.9	4.1	115.4	1.7
3，4-DBP	85.7	2.0	84.2	2.2	99.8	2.1
OMBB	112.3	5.3	91.9	4.8	109.8	2.0
EHDAB	118.0	2.4	118.9	2.0	100.7	1.0
EDB	118.5	1.7	119.5	1.7	107.6	1.2
2-ITX	119.0	2.4	119.7	4.2	97.6	2.3
DETX	113.9	3.2	93.2	1.2	102.4	2.3
DEAB	86.3	4.7	89.6	5.2	114.9	2.6
MK	104.6	5.6	109.0	4.7	108.0	2.1

### 2.6 实际样品测定

采用建立的UPC^2^-MS/MS方法对12批药用复合膜样品中的光引发剂进行检测，外标法定量，结果见表5，有3批样品检测到光引发剂4-MBP，有4批样品检测到光引发剂OMBB，1批样品检测到光引发剂BP、2-ITX。参考光引发剂BP口服途径的允许日暴露量（permitted daily exposure，PDE），12种光引发剂的PDE都以2500 μg/d计算^［6］^，并以药用复合膜每日最大使用量600 cm^2^计算^［25］^，将光引发剂的分析评价阈值（analytical evaluation threshold，AET）拟为4.2 mg/cm^2^。本实验收集到的药用复合膜中的光引发剂最高检出含量是0.12 μg/cm^2^，未超过拟定限度。

**表5:** 阳性药用复合膜样品中光引发剂检测的结果 (μg/cm^2^)

Compound	Y03	Y04	Y05	Y06	Y08	Y10
BP	-	-	-	-	-	0.03
4-MBP	-	-	0.08	0.07	0.09	-
OMBB	0.03	0.03	-	--	0.12	0.11
2-ITX	-	-	-	-	-	0.04

-： not detected （<LOD）.

## 3 结论

本实验建立的UPC^2^-MS/MS方法灵敏度高，分析效率高，经济环保，适用于监测药用复合膜中光引发剂的残留量，能为药用复合膜的选择提供新方法工具，也为日后药品包装材料质量标准的完善提供了研究基础。
